# Dietary Restriction Affects Neuronal Response Property and GABA Synthesis in the Primary Visual Cortex

**DOI:** 10.1371/journal.pone.0149004

**Published:** 2016-02-10

**Authors:** Jinfang Yang, Qian Wang, Fenfen He, Yanxia Ding, Qingyan Sun, Tianmiao Hua, Minmin Xi

**Affiliations:** 1 College of Life Sciences, Anhui Normal University, Wuhu, Anhui, China; 2 Business School, University of the West Scotland, Glasgow, United Kingdom; Centre de Neuroscience Cognitive, FRANCE

## Abstract

Previous studies have reported inconsistent effects of dietary restriction (DR) on cortical inhibition. To clarify this issue, we examined the response properties of neurons in the primary visual cortex (V1) of DR and control groups of cats using *in vivo* extracellular single-unit recording techniques, and assessed the synthesis of inhibitory neurotransmitter GABA in the V1 of cats from both groups using immunohistochemical and Western blot techniques. Our results showed that the response of V1 neurons to visual stimuli was significantly modified by DR, as indicated by an enhanced selectivity for stimulus orientations and motion directions, decreased visually-evoked response, lowered spontaneous activity and increased signal-to-noise ratio in DR cats relative to control cats. Further, it was shown that, accompanied with these changes of neuronal responsiveness, GABA immunoreactivity and the expression of a key GABA-synthesizing enzyme GAD67 in the V1 were significantly increased by DR. These results demonstrate that DR may retard brain aging by increasing the intracortical inhibition effect and improve the function of visual cortical neurons in visual information processing. This DR-induced elevation of cortical inhibition may favor the brain in modulating energy expenditure based on food availability.

## Introduction

An increasing body of evidence indicates that dietary restriction (DR) or calorie restriction can significantly extend lifespan in diverse species from yeast to primates including humans [1–6]. Therefore, DR has been widely accepted as a potential noninvasive anti-aging therapy [7–10]. Several observations have found that DR can stimulate production of neurotrophic factors [11–13], modify brain plasticity [14–18], retard age-related neurodegeneration and decline in learning and memory [19, 20]. Therefore, DR may exert protective effects on brain during senescence [15, 21] and thus mediate the lifespan extension.

Recent investigations on brain aging, especially on the sensory cortex, indicated that a reduction of intracortical inhibition may underlie neuronal function degradation during senescence [22–28]. This age-dependent decrease of inhibition is closely related to a reduction of GABA synthesis [29, 30]. If DR can protect the brain from aging, how does it affect intracortical inhibition and GABA synthesis? Answers to this question are at present diverse. Spolidoro et al. [17] reported that DR in adult rats was able to reinstate ocular dominance plasticity in the visual cortex and promote recovery from amblyopia, and these effects were accompanied by a reduction of intracortical inhibition. However, other research groups found that DR in adult animals could significantly increase the GAD expression [31, 32] and GABA production in the brain [13], suggesting a DR-induced elevation of cortical inhibition. Still others reported no significant changes of GABA in the brain under dietary protein restriction [33, 34].

If DR modifies the strength of intracortical inhibition, it can be predicted that the response property of cortical neurons will change with DR. To test this possibility, we reared 4 adult cats with 30% DR for 3 months and 4 adult cats with food *ad libitum* as controls. At the end of DR period, we examined the neuronal responsiveness in the primary visual cortex (V1), attempting to see if DR could affect the function of visual cortical neurons. Additionally, the GABA-immunoreactive intensity and the expression of GAD67 (a key GABA-synthesizing enzyme, glutamic acid decarboxylase) in the V1 of both DR and control groups were also measured to assess whether the synthesis of inhibitory GABA neurotransmitters altered with DR.

## Materials and Methods

### Subjects and food restriction manipulation

Eight adult female cats used in this study were purchased (age: 2 years old; body weight: 2.8–3.5 kg) from Nanjing Qing-Long-Shan Animal Breeding Farm (Jiangning District of Nanjing city, Certificate No. SX1207) and reared in our laboratory for about 2 years to accommodate to new surroundings and the experimenters. They were pathogen-free, disease-free healthy subjects as indicated by medical examinations from veterinarians. All subjects had no optical or retinal problem that would impair their visual functions and had never been used in previous experiments. Each individual cat was housed in a small room (2 m × 2 m ×2.7 m) separated by transparent glass walls. Each room had comfortably organized living, feeding and playing areas, and room temperature was kept at 25°C. Cats could get water and food freely and play toys, such as moving rats and frogs. Furnishings in the room were cleaned every day and sterilized regularly.

Before DR manipulation, all cats (age: 4–5 years old; body weight: 3.4–3.8 kg) were allowed to get food (containing 26% protein, 9% fat and 41.2% carbohydrate) freely for 1 week so that we could measure the normal average amount of daily diet for each cat. Subsequently, eight cats were randomly divided into two groups, with 4 cats in each group. One group was used as the DR group, and each cat received 70% of normal daily diet, a regime that has been applied previously in different animal species [2, 4, 6, 13, 20, 35]. Another group of cats were used as controls and could freely get food. DR lasted for 3 months, and their body weights were monitored on a weekly basis. To assess the animals’ health condition, their body temperature (38–38.5°C), heart rate (180–220 pulses/min), femoral artery blood pressure (100–130 mm Hg / 30–40 mm Hg) and blood oxygen saturation (SpO_2_ ≥ 94%) were measured non-invasively. We set a maximum weight loss threshold within 25% at which the animals would be euthanized by stopping its breath and heart beat through intravenous injection of pentobarbital sodium (> 100 mg/kg).

Experimental procedures in this study were performed strictly in accordance with the Guide for the Care and Use of Laboratory Animals of the National Institutes of Health. All animal treatments in this research were approved by the Ethics Committee of Anhui Normal University, and all efforts were made to minimize suffering or distress.

### Extracellular single-unit recording and data analysis

#### Preparation for single-unit recording

Each cat was prepared for acute *in vivo* single-unit recording after at the end of DR period. The recording procedures were similar to that described in our previous studies [24, 36–38]. Briefly, anesthesia was induced by injection of ketamine HCl (40 mg/kg, im) and xylazine (2 mg/kg, im). After intubation of intravenous and tracheal cannulae, the cat was immobilized in a stereotaxic apparatus with ear, eye and bite bars. Pupils were maximally dilated with atropine (1%) eye drops, and plano contact lenses were used to protect the corneas. Neosynephrine (5%) was applied to retract the nictitating membranes. Glucose (5%)-saline (0.9%) solution containing urethane (20 mg/hr/kg body weight) and gallamine triethiodide (10 mg/hr/kg body weight) was infused intravenously by a syringe pump to keep the animal anesthetized and paralyzed. Artificial respiration was performed, and expired pCO_2_ was maintained at approximately 3.8%. Heart rate (approximately 180–220 pulses/min) and electrocardiogram (ECG) were monitored throughout the electrophysiology experiment in order to assess the level of anesthesia and ensure the animals were not experiencing pain.

The primary visual cortex (V1) was partly exposed (8 mm posterior to the ear bar, 4 mm lateral to the midline) by removing the skull and dura over V1 (area 17) with the aid of a surgery microscope. The small hole over V1 was filled with 4% agar saline solution prior to electrophysiological recording. The optic discs of the two eyes were reflected onto a movable transparent tangent screen positioned 57 cm from the animal’s eyes and overlapped with a CRT monitor (resolution 1024×768, refresh rate 85 Hz) for presentation of visual stimuli. The retinal central area of each eye was precisely located according to the position of the optic discs reflected onto the tangent screen [39]. After all the preparations were completed, single-unit recordings were performed using a glass-coated tungsten microelectrode (with an impedance of 3–5 MΩ) which was advanced by a hydraulic micromanipulator (Narishige, Japan). When the experiment was complete, the distance of each recorded cell’s receptive field from the retinal central area was measured and calculated as visual angle.

#### Visual stimuli

Visual stimuli consisted of moving sinusoidal gratings, which were generated in MATLAB with the aid of extensions provided by the high-level Psychophysics Toolbox [40] and low-level Video Toolbox [41]. Once a cell’s visually-evoked response was detected, the cell’s receptive field center was preliminarily determined using bars of light emitted from a hand pantoscope and then precisely mapped by presenting repeatedly a series of computer-generated flashing bars of light on the CRT. We selected optimal stimulus size, temporal and spatial frequency for each cell. Each stimulus was presented to the dominant eye. Then, a set of grating stimuli with optimal stimulus parameters, moving in 24 different directions (0–360° scale with an increment of 15°) was used to compile the orientation and direction tuning curves. The orientation of each drifting stimulus was orthogonal to its direction of motion. Each stimulus was presented repeatedly 4–6 times. Before each stimulus presentation, the baseline response (spontaneous activity) was obtained while mean luminance was shown on the display for 1s. The duration of each stimulus presentation was less than 5s with a 2 min interval between stimuli for the cell’s functional recovery. The contrast for each stimulus was set at 100%. The mean luminance of the display was 19 cd/m^2^, and the environmental luminance on the cornea was 0.1 lx.

#### Data collection and analysis

Action potentials of recorded cells were amplified with a microelectrode amplifier (Nihon Kohden, Japan) and differential amplifier (Dagan 2400A, USA), and then fed into a window discriminator with an audio monitor. The original voltage traces were digitized by an acquisition board (National Instruments, USA) controlled by IGOR software (WaveMetrics, USA), and saved for on- or off-line analysis. A cell’s response to a grating stimulus was defined as the mean firing rate (spontaneous response subtracted) corresponding to the time of stimulus presentation, which was used to acquire the curves of tuning response to stimulus orientations, temporal and spatial frequencies.

**Fig 1:**
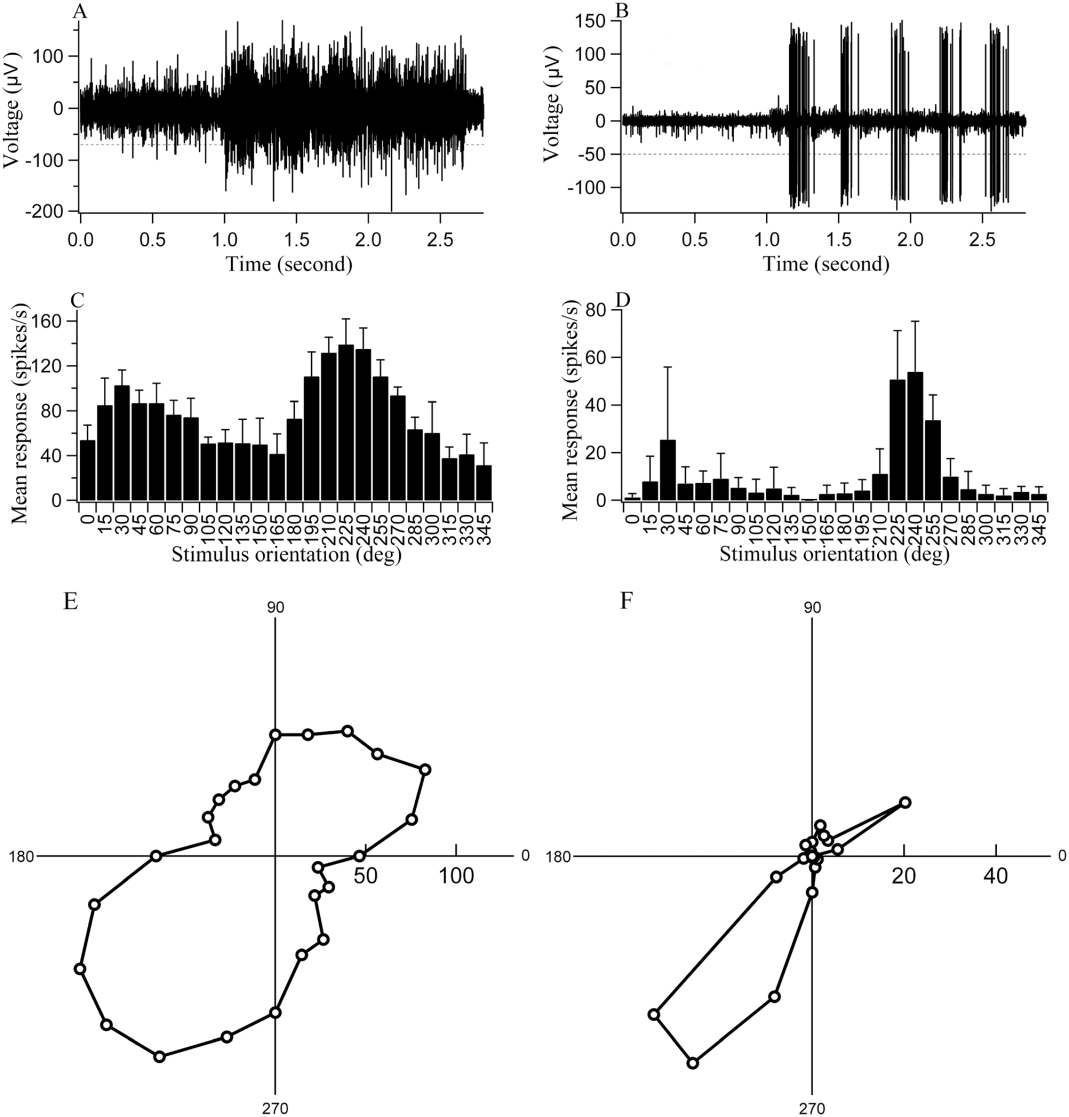
The response property of two typical neurons from the normal control (A, C, E) and DR (B, D, F) group of cats respectively. (A&B) The voltage trace of the neuron’s response to its preferred stimulus orientation and motion direction. A spike with amplitude above the horizontal broken line was counted as an action potential. Spontaneous activity was acquired during 1s pre-stimulus period. The neuron’s visually-driven response was evoked by 5 cycles of drifting grating stimulus with the preferred orientation, equivalent to a stimulus duration of 1.7s. (C&D) Mean response (pole with error bar) of the neuron to different stimulus orientations. The maximum response represented the neuron’s response to the preferred stimulus orientation and motion direction. (E&F) Circle variance showed the neuron’s response selectivity for stimulus orientations and motion directions, with orientation bias (OB) of 0.275 and 0.747 respectively, motion direction bias (DR) of 0.133 and 0.487 respectively.

The preferred orientation, orientation bias and motion direction bias for each cell were obtained as previously described [22, 24, 37, 38]. Briefly, the responses of each cell to the different stimulus orientations or directions were stored as a series of vectors. The vectors were added and divided by the sum of the absolute values of the vectors. The angle of the resultant vector gave the preferred orientation or motion direction of the cell. The length of the resultant vector, termed the orientation or motion direction bias (OB or DB), provided a quantitative measure of the orientation or direction sensitivity of the cell (Fig 1). A cell’s signal-to-noise ratio (STN) was defined as the ratio between the cell’s visually evoked response to the optimal stimulus and the cell’s baseline response. To avoid data skewing or overestimation, all baseline response below 1 spike/s were set equal to 1 spike/s for the signal-to-noise ratio calculation.

Statistical comparisons between the DR and the control groups of cats were carried out using one or two-way ANOVA. All mean values were expressed as mean ± standard deviation.

### Immunohistochemical labeling and Western blotting

At the end of electrophysiological single-unit recording, the V1 (area17) on one cerebral hemisphere was completely exposed by removing the overlaid skull. After the cat was deeply anesthetized with ketamine HCl (80 mg/kg, im) and xylazine (4 mg/kg, im), the exposed unilateral V1 was quickly removed and frozen with liquid nitrogen, which was then stored at -70°C until preparation for Western blot assays. Immediately after removal of the exposed brain tissue, the cat was transcardially perfused with 500 ml saline solution (0.9%) followed by 100 ml fixative solution containing 2% paraformaldehyde. Then, brain tissue containing V1 on another hemisphere was dissected and post-fixed in 4% paraformaldehyde (containing 15% sucrose) at 4°C for 24h, which was used for sectioning and immunohistochemical labeling.

#### GABA-immunohistochemical labeling

Post-fixed V1 tissue was transferred to 30% sucrose and stored at 4°C until tissue sinking. Frozen sections (thickness of 30 μm) were mounted on gelatin-coated glass slides. From each animal, 10 sections were sampled (at an interval of about 400 μm apart) for Nissl staining. Two adjacent sections were used for immunohistochemical labeling of GABAergic neurons and immunoreaction control.

Antiserum to GABA (rabbit polyclonal; 1:1500; Lab Visio Corporation) was applied to visualize GABA-immunoreactive neurons in the visual cortex. Sections were first rinsed in 0.1M PBS (pH 7.4) for 10 min, and then incubated with 0.3% H_2_O_2_ in PBS for 15 min to quench endogenous peroxidase activity. Following washing in PBS (3×10 min), the sections were incubated with 5% normal goat serum in PBS for 10 min at room temperature to block non-specific reactions. Subsequently, the sections were incubated with primary antibody against GABA for 24h at 4°C, washed in PBS (3×10 min) and then incubated with biotinylated goat anti-rabbit IgG for 10 min at room temperature. After further rinsing in PBS (3×10 min), the sections were incubated at room temperature with an ABC solution (including 10 min of treatment with streptavidin peroxidase, 10 min of rinsing in PBS and then 10 min of incubation with a mixture of DAB chromogen and DAB substrate). After rinsing in PBS, dehydrating in gradient alcohol and clearing in xylene, the sections were finally coverslipped with Permount. Control sections were stained simultaneously following the same procedure as described above with the exception that the primary antibody was replaced by PBS. We used an optimal dilution (1:1500) of anti-GABA serum for GABAergic neurons visualization. Nissl staining (0.5% thionine 37°C, 40 min) was used for identification of V1 cortical layers.

Nissl-stained and GABA-immunoreactive slices were observed under a microscope (Olympus BX-51). Images were collected by a high resolution (5,000,000 pixels) digital camera controlled by Image-Pro Express 6.0 software. Forty image samples (each with an area of 100 × 100 μm^2^) were randomly selected from each slice, and their optical density (OD) value were measured with the background calibrated using a batch-measure function of Image-Pro-Plus 6.0 [42–44]. The average OD value was taken as the index indicating the intensity of GABA immunoreactivity. All data were expressed as mean ± standard deviation and analyzed via ANOVA or T-test, with *P*<0.05 being considered statistically significant.

#### Western blot preparation and ELISA

Western blots were performed as described previously [45–47]. In brief, frozen V1 tissues were cut, weighed, thawed, and homogenized in 10 volumes of an ice-cold buffer (25 mM Tris–HCl pH 7.6; 150 mM NaCl, 1% NP-40, 1% sodium deoxycholate and 0.1% SDS) and a protease inhibitor cocktail (Kang Chen Biotechnology, Shanghai, China) and spun down at 12,000 rpm for 15 min at 4°C. Protein concentration in the supernatant was measured using Coomassie brilliant blue G-250 (Sangon Biotechnology, Shanghai, China). Proteins (50 μg) from each sample of different individual cats were fractionated using 8% sodium dodecyl sulfate polyacrylamide gel electrophoresis (SDS-PAGE) and transferred onto polyvinylidene fluoride (PVDF) membranes (Beyotime Biotechnology, Shanghai, China). The membrane was blocked with 5% non-fat dry milk in TBS-Tween 20 for one hour and incubated overnight at 4°C in TBS-Tween 20 containing a rabbit polyclonal antibody GAD67 (dilution 1:200; Boster Bio-engineering Limited Company) or a rabbit polyclonal antibody GAPDH (glyceraldehyde-3-phosphate dehydrogenase) (dilution 1:5,000; Sangon Biotechnology). The membranes were washed 3 times for 5 min with TBS-Tween 20 and incubated with Peroxidase-conjugated AffiniPure Goat Anti-Rabbit IgG (AB10058, Sangon Biotechnology, China), dilution 1:5,000 in TBS-Tween-20 for 2 h at 25°C and washed again in TBS–Tween-20. Then the membranes were developed with BeyoECL Plus (Beyotime Biotechnology, Shanghai, China) and the signal visualized on Kodak X-OMAT LS film (Sigma). The optical density (OD) of Western blot bands was measured using Image J software. The OD value of GAD67 band was expressed relatively to the corresponding GAPDH band from the same sample.

To exclude the possibility that the expression of internal reference GAPDH was modified by DR, the concentration of GAPDH protein in the total proteins extracted from V1 tissues of each control and DR cat was measured using enzyme linked immunosorbent assay (ELISA) kit for rat GAPDH (S1 Text).

## Results

### Body weight changes during DR

**Fig 2:**
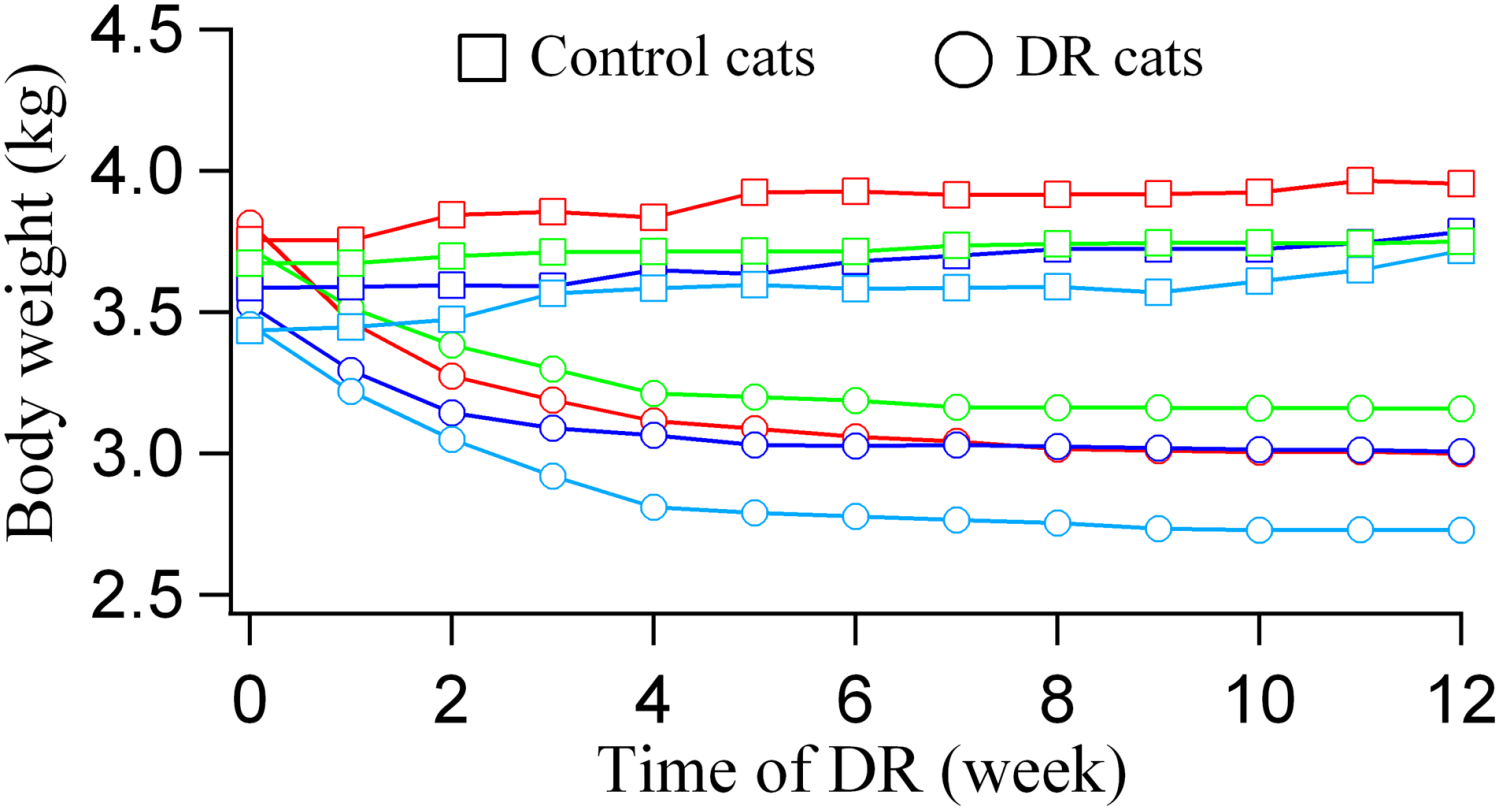
Body weight (BW) changes of each DR cat (open circle) and normal control cat (open square) during the period of DR. The BW of normal control cats (NC1: red; NC2: blue; NC3: cyan; NC4: green) showed an insignificant increase (p = 0.07), whereas the BW of DR cats (DR1: red; DR2: blue; DR3: cyan; DR4: green) were significantly decreased (p<0.01).

**Fig 3:**
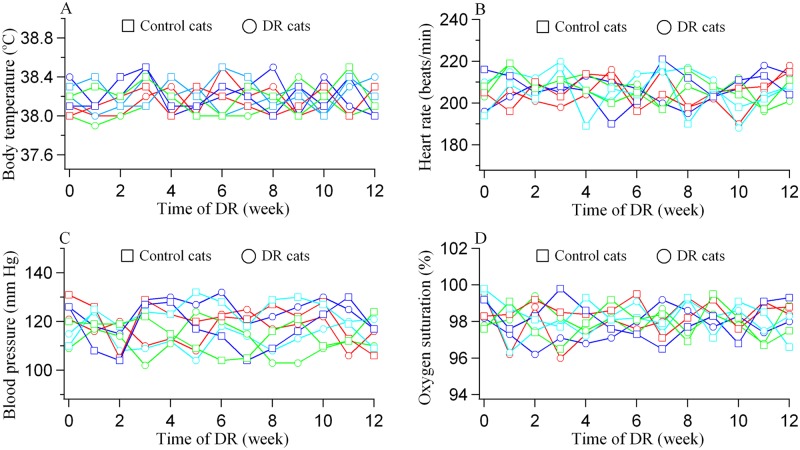
Record of body temperature (A), heart rate (B), femoral artery contraction blood pressure (C) and blood oxygen saturation (D) for each DR cat and control cat during the period of DR. Open squares represented normal control cats (NC1: red; NC2: blue; NC3: cyan; NC4: green). Open circles denoted DR cats (DR1: red; DR2: blue; DR3: cyan; DR4: green).

We monitored the body weight (BW) of each cat weekly during the period of DR. The BW of cats in the normal control group (NC1-4) showed a small increase. However, the BW of cats in DR group (DR1-4) decreased evidently during 3 months of DR (Fig 2 and S1 Table). Relative to before DR experiment, the BW of NC1, NC2, NC3 and NC4 after experiment increased by 5.3%, 5.5%, 8.3% and 2.1% respectively, whereas the BW of DR1, DR2, DR3 and DR4 at the end of DR period reduced by 21.2%, 14.5%, 21.0% and 15.3% respectively. This level of weight loss was anticipated according to findings of previous studies [13, 48–52] and detailed in the study protocol submitted for review and approval by the ethics committee. Although DR cats, especially DR1 and DR3, had a weight loss approaching 21%, they all showed a normal behavior in daily activities, and their body temperature, heart rate, blood pressure and blood oxygen saturation during DR were in the normal range (Fig 3 and S2 Table) and exhibited no significant difference from that of normal control cats (all p>0.1). Additionally, the DR-induced BW reduction mainly occurred during the first 4 weeks of DR (Fig 2), and their BW stabilised at the reduced level during the 2^nd^ and 3^rd^ months. This may mean that the energy metabolism of the DR cats showed an adaptation to the food shortage after the first 4 weeks.

### DR affects response property of V1 neurons

To confirm if DR affected the function of V1 neurons in visual signal processing, we examined the response of V1 neurons to visual stimuli with different orientations and motion directions. 88 neurons in the normal control cats (NC1-4) and 75 neurons in DR cats (DR1-4) were studied in this study (Table 1). All neurons had a receptive field within 8° visual angle from the retinal central area of dominant eye.

**Table 1:** Measures of the cell number (CN), mean orientation bias (OB), motion direction bias (DB), maximum response (MR) to the preferred stimulus orientation, average response (AR) to all stimulus orientations, baseline response (BR) and signal-to-noise ratio (STN) for studied neurons of each normal control cat (NC1, NC2, NC3, NC4) and DR cat (DR1, DR2, DR3, DR4).

Subject	CN	OB	DB	MR	AR	BR	STN
**NC1**	19	0.26±0.11	0.15±0.10	62.9±18.1	28.2±9.1	8.1±5.6	10.6±6.0
**NC2**	23	0.26±0.12	0.11±0.12	65.9±20.2	29.3±9.6	9.9±7.1	13.3±13.8
**NC3**	21	0.23±0.18	0.14±0.11	70.6±29.1	35.8±18.2	9.5±6.9	13.4±11.8
**NC4**	25	0.26±0.19	0.13±0.10	66.1±28.1	31.8±19.6	10.4±7.3	13.5±14.9
**DR1**	17	0.53±0.15	0.36±0.15	45.9±12.7	13.1±7.5	2.4±1.1	22.4±10.1
**DR2**	17	0.39±0.20	0.31±0.26	42.9±24.8	15.3±11.7	3.0±3.0	29.2±29.1
**DR3**	16	0.48±0.23	0.26±0.21	48.2±21.1	14.9±9.6	3.4±4.5	31.7±21.7
**DR4**	26	0.41±0.20	0.24±0.15	45.6±19.5	11.9±3.7	2.7±1.9	25.0±18.7

#### Response selectivity of V1 neurons

**Fig 4:**
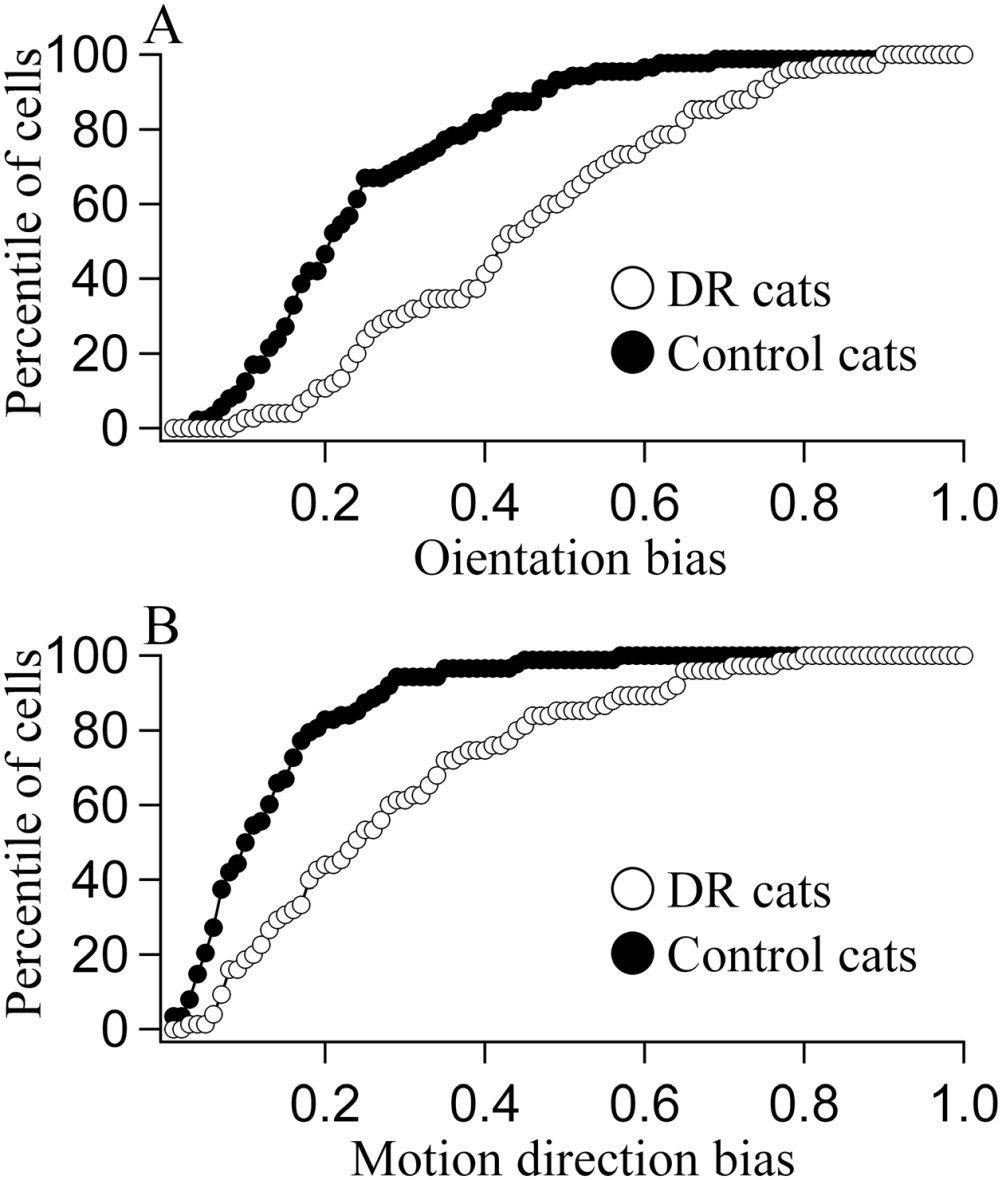
Percentile value of neurons showing different orientation bias (OB) (A) and motion direction bias (DB) (B) for DR cats (open circle) and normal control cats (solid circle). The total number of neurons was 75 and 88 respectively for DR cats and control cats. A percentile value indicated the percentage of neurons whose OBs or DBs were lower than the corresponding OB or DB value on the horizontal axis. DR cats showed significantly increased OB and DB value compared with control cats (p<0.0001; p<0.0001).

We first compared the selectivity of V1 neurons for different stimulus orientations and motion directions. The majority of neurons (69.3%) in the control group of cats had an orientation bias (OB) value less than 0.3, whereas 68% neurons in DR group had an OB value larger than 0.3 (Fig 4A). As indicated by ANOVA analysis, the mean OB within either control or DR group showed no significant difference between individual cats (Control group: F(3,88) = 0.196, p>0.5; DR group: F(3,75) = 1.681, p>0.1). However, the mean OB of each individual cat in the DR group was significantly larger than that of any individual cat in the control group (Group effect: F(1,163) = 48.206, p<0.0001; Interaction of group and individual cat: F(3,163) = 1.351, p>0.05). Additionally, the average OB value across all cats in the DR group (0.45 ± 0.20) was also significantly higher than in the control group (0.25 ± 0.16) (F(1,163) = 46.725, p<0.0001). Similarly, most of neurons (80.6%) in the control group of cats had a motion direction bias (DB) value smaller than 0.2, whereas more than half (56%) of neurons in the DR group had a DB value higher than 0.2 (Fig 4B). The mean DB within the control or DR group exhibited no significant difference between cats (Control group: F(3,88) = 0.363, p>0.5; DR group: F(3,75) = 1.315, p>0.1), whereas the mean DB of each cat in the DR group was significantly larger than that of any individual cat in the control group (Group effect: F(1,163) = 42.705, p<0.0001; Interaction of group and individual cat: F(3,163) = 1.149, p>0.1). The average DB of all cats in the DR group (0.29±0.19) was also significantly higher than that in the control group (0.13±0.10) (F(1,163) = 41.187, p<0.0001). Therefore, we concluded that DR increased the selectivity of V1 neurons for stimulus orientations and motion directions.

#### Visually-evoked response and spontaneous activity

The enhanced selectivity of V1 neurons for visual stimulus orientations and motion directions in DR cats could result from an increased response to the optimal stimulus orientation or a decreased response to non-optimal orientations. To clarify this possibility, we compared the neuronal maximum response to its preferred stimulus orientation and the average response to all stimulus orientations between DR group and the control group.

**Fig 5:**
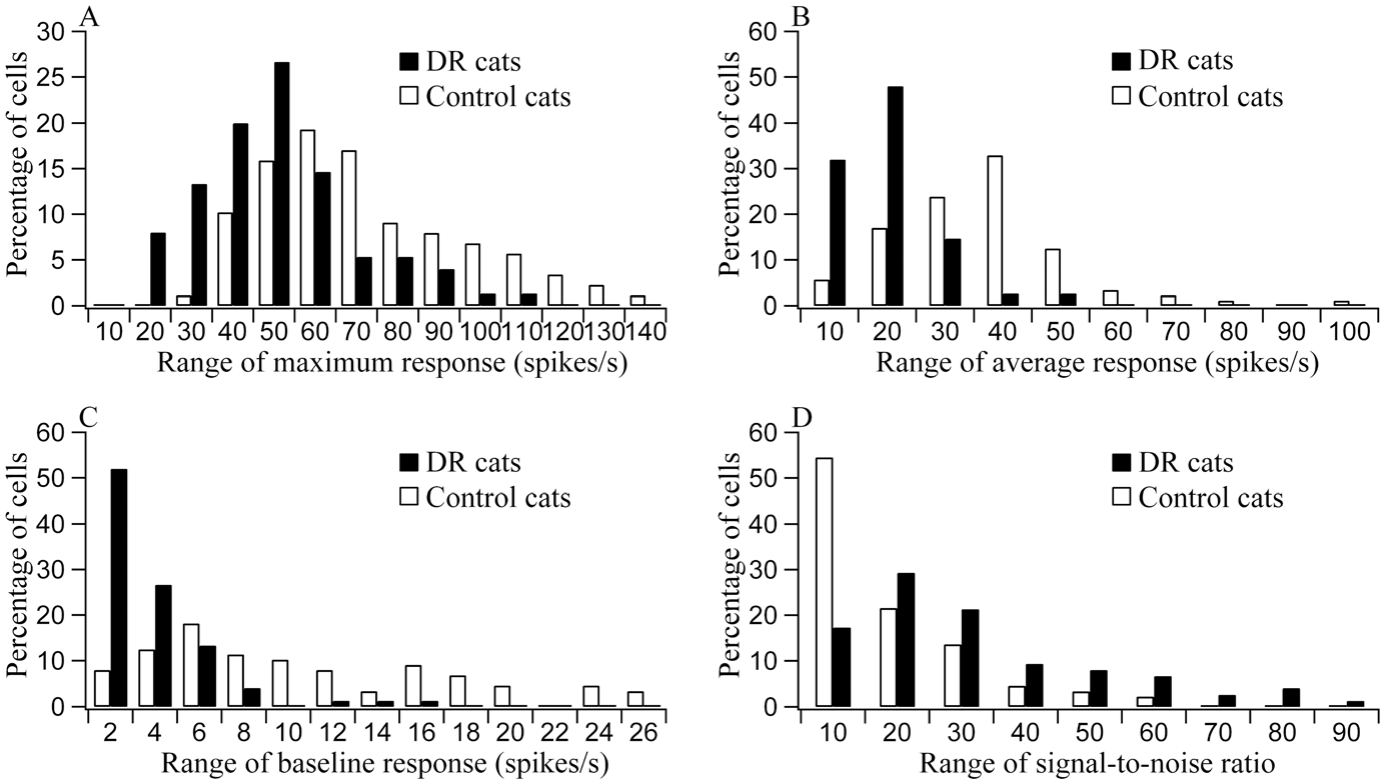
Percentage of neurons with different range of maximum response (MR) (A), average response (AR) (B), baseline response (BR) (C) and signal-to-noise ratio (STN) (D) for DR and normal control cats. The total number of neurons was 75 and 88 respectively for DR cats and control cats. DR cats showed significantly lower MR, AR and BR (all p<0.0001), but significantly higher STN (p<0.0001) when compared with control cats.

Most of neurons (72.7%) in the control group showed a maximum response (MR) larger than 50 spikes/s, whereas 68% neurons in the DR group displayed a MR smaller than 50 spikes/s (Fig 5A). ANOVA analysis indicated that the mean MR was not significantly different between cats within either DR group or control group (Control group: F(3, 88) = 0.337, p>0.5; DR group: F(3, 75) = 0.189, p>0.5). However, the mean MR value of each individual cat in the DR group was significantly lower when compared with that of any individual cat in the control group (Group effect: F(1, 163) = 33.528, p<0.0001; Interaction of group and cat: F(3, 163) = 0.133, p>0.5). The average MR value across all cats in the DR group (45.6 ± 19.6) was also significantly lower than in the control group (66.4 ± 24.2) (F(1, 163) = 35.557, p<0.0001). Relative to the control group of cats, the average MR in the DR group was lower by 31.3%.

Most of the observed neurons (77.2%) in the control group had an average response (AR) higher than 20 spikes/s, whereas most of neurons (80%) in the DR group had a AR less than 20 spikes/s (Fig 5B). The mean AR displayed no significant difference between cats in either DR or control group (Control group: F(3, 88) = 1.01, p>0.1; DR group: F(3, 75) = 0.715, p>0.5). Nevertheless, the mean AR of each cat in the DR group was significantly lower when compared with that of any individual cat in the control group (Group effect: F(1, 163) = 77.23, p<0.0001; Interaction of group and cat: F(3, 163) = 0.732, p>0.5). The averaged AR of all cats in the DR group (13.6 ± 8.1) was also significantly lower than in the control group (31.3 ± 15.2) (F(1, 163) = 81.592, p<0.0001). Relative to the control group of cats, the average AR in the DR group was lower by 56.5%. Therefore, DR decreased the response of V1 neurons at all stimulus orientations, but the amplitude of the decrease at non-optimal orientations was greater than at the optimal orientation.

To examine whether the spontaneous activity (BR: baseline response) of V1 neurons was also modified by DR, we compared the mean BR value between the two groups of cats. More than half of the neurons (61.3%) in the control group of cats had a BR greater than 6 spikes/s, whereas the majority of the neurons (78.6%) in the DR group had a BR fewer than 4 spikes/s (Fig 5C). ANOVA analysis showed that the average BR of all cats in the DR group (2.8 ± 2.7) was significantly lower than in the control group (9.5 ± 6.7) (F(1, 163) = 65.062, p<0.0001). Further, the mean BR of each cat in the DR group was significantly smaller than that of any individual cat in the control group (Group effect: F(1, 163) = 60.227, p<0.0001; Interaction of group and cat: F(3, 163) = 0.279, p>0.5). Relative to the control group of cats, the average BR in the DR group was 70.3% lower. Because of a large reduction of BR, the signal-to-noise ratio (STN) of V1 neurons in the DR group was significantly higher than that in the control group. The majority of neurons (76.1%) in the control group of cats had a STN value smaller than 20, whereas more than half of neurons (53.3%) in the DR group had a STN higher than 20 (Fig 5D). The averaged STN in the DR group (26.7 ± 20.6) was significantly larger than in the control group (12.8 ± 12.2) (F(1, 163) = 28.446, p<0.0001). Further, the mean STN of each cat in the DR group was significantly higher than of any individual cat in the control group (Group effect: F(1, 163) = 28.932, p<0.0001; Interaction of group and cat: F(3, 163) = 0.377, p>0.5).

All the above analysis demonstrated that DR depressed both spontaneous activity and the visually-evoked response of the V1 neurons, but increased their selectivity and signal-to-noise ratio in response to visual stimuli.

### DR affects GABA synthesis

To assess if DR affected the synthesis of inhibitory neurotransmitters, we measured the GABA-immunoreactive intensity and the expression of a key subunit of GABA-synthesizing enzyme GAD67 in V1 of cats from DR and control groups.

**Fig 6:**
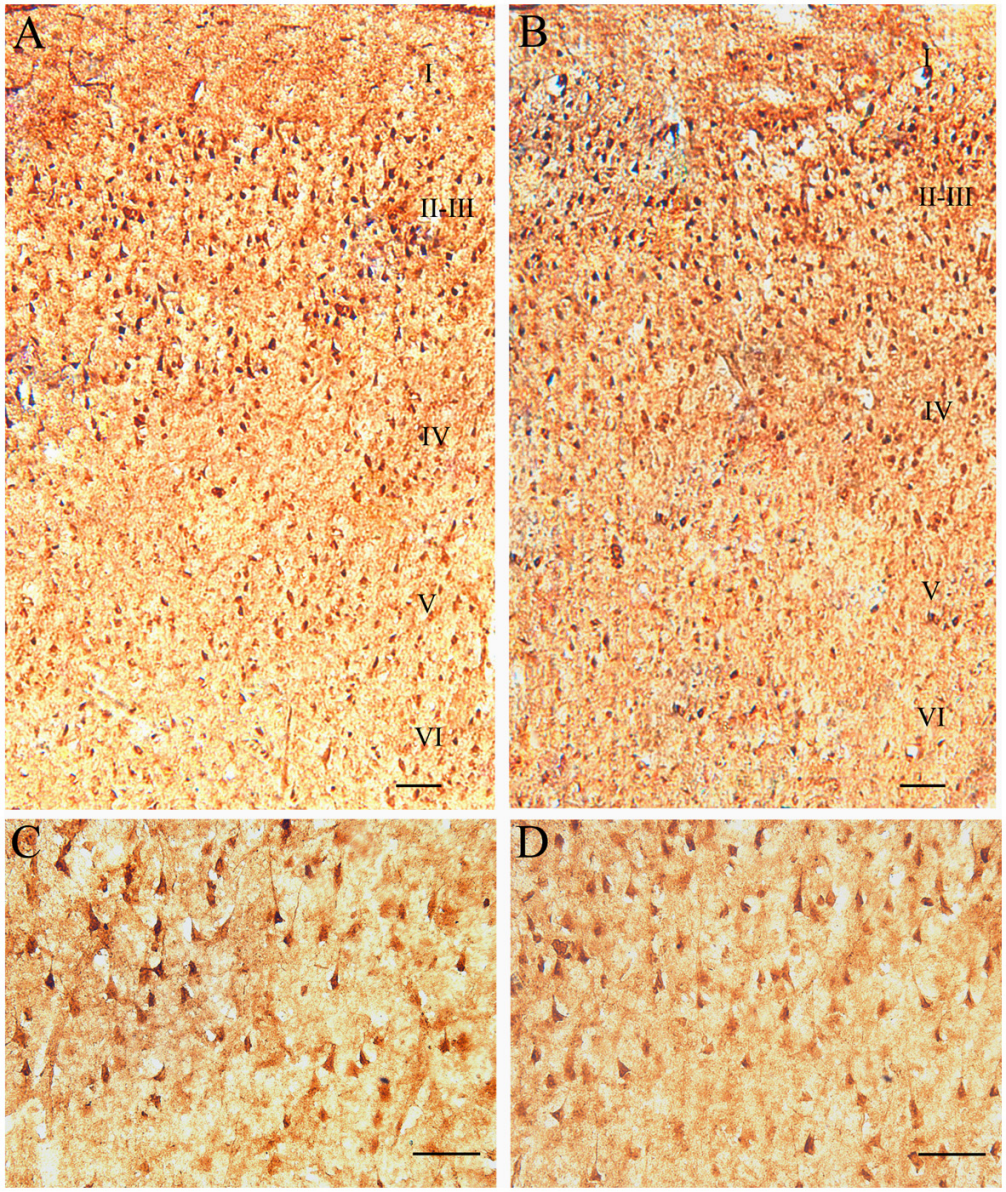
Immunohistochemical labeling of GABAergic neurons in the primary visual cortex of DR cats (A&C) and normal control cats (B&D). (A&B) show the distribution of GABA neurons across different cortical layers (layer I, II-III, IV, V and VI) at a low amplification. (C&D) show GABA neurons at a higher amplification. The scale bar equals to 25 μm.

GABA-immunoreactive neurons, as indicated by brown or dark brown-colored GABA-positive substance in the somatic cytoplasm, were seen at all cortical layers in both control and DR groups of cats. GABA-immunoreactive neurons were quite sparse and small in the layer I, but denser and larger in the layers II-III, IV, V and VI, with the highest density in the layer II-III (Fig 6).

**Fig 7:**
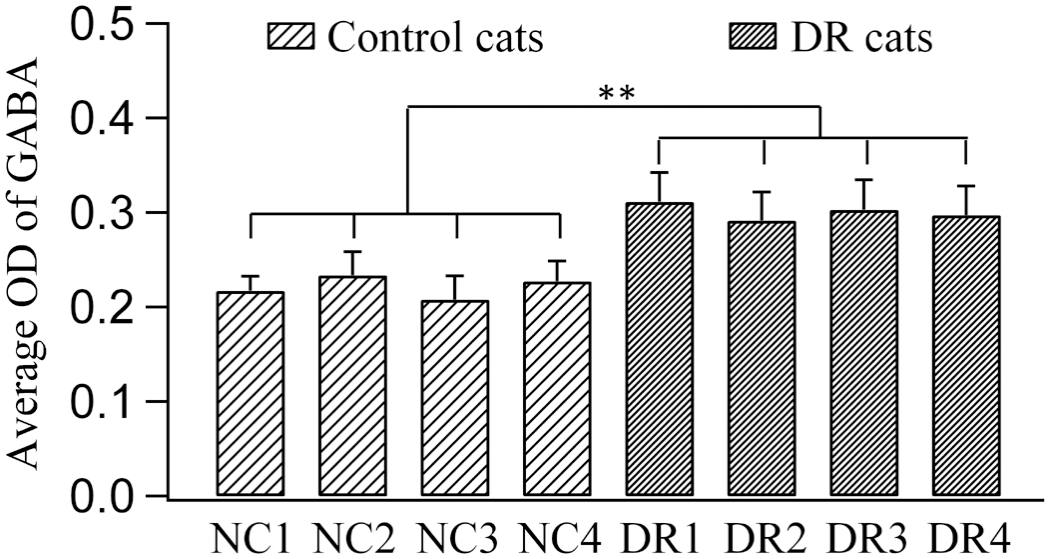
Poles with error bars represented the average optical density (OD) value of GABA immunoreactivity in the primary visual cortex of each normal control cat (NC1-4) and DR cat (DR1-4). The mean OD of GABA immunoreaction in each DR cat was significantly larger than that in any individual control cat (** p<0.001).

ANOVA analysis indicated that the mean optical density (OD) of GABA immunoreaction showed no significant difference between individual cats within either control group (F(3,40) = 1.392, p>0.1) or DR group (F(3,40) = 0.753, p>0.5). However, the mean OD of GABA immunoreaction in each DR cat was significantly larger than that in any individual control cat (Group effect: F(1,80) = 164.349, p<0.0001; Interaction of group and subject: F(3,80) = 1.125, p>0.1) (Fig 7). Further, the averaged OD value of GABA immunoreaction across all cats in DR group (0.30 ± 0.031) was also significantly higher than that in control one (0.22 ± 0.022) (F(1,80) = 164.7, p<0.0001).

**Fig 8:**
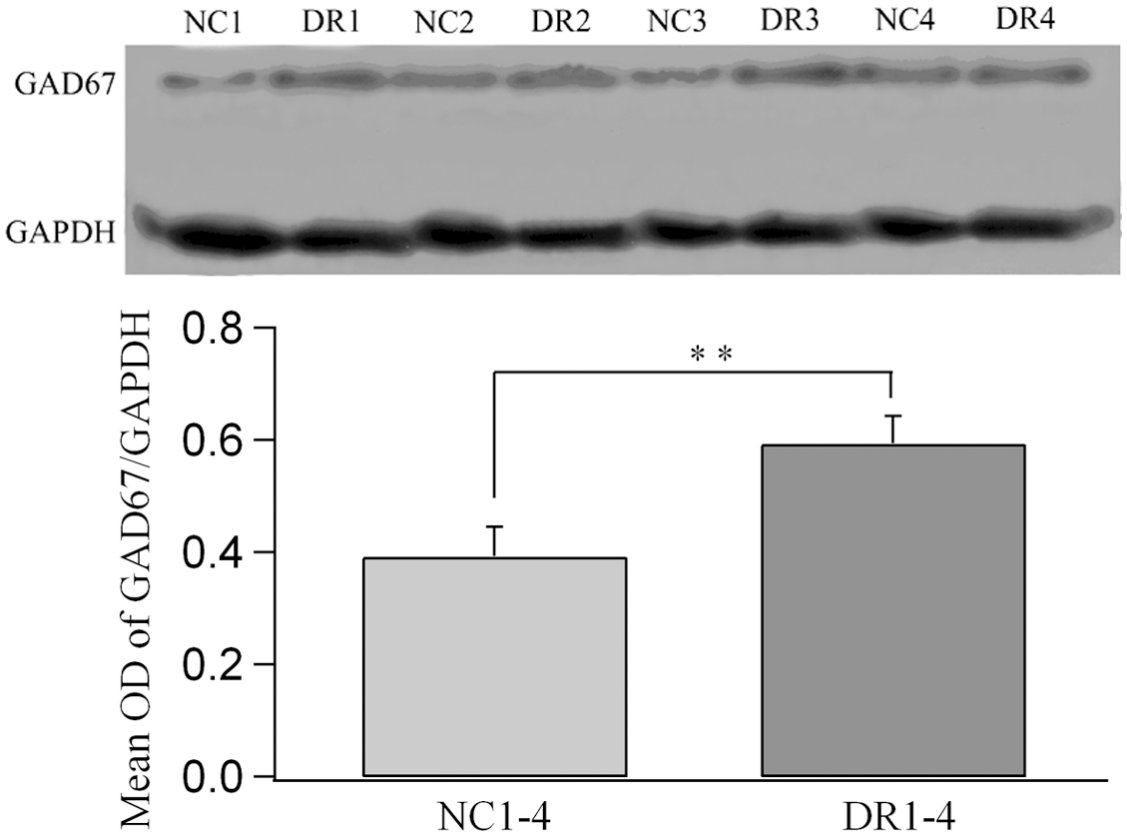
Comparison of the expression levels of GABA-synthesizing enzyme subunit, GAD67, between DR and normal control cats. The top panel shows a typical sample of a Western blot result from each DR cat (DR1-4) and control cat (NC1-4) using GAD67 and GAPDH (glyceraldehyde-3-phosphate dehydrogenase) antibodies. The bottom panel shows the average optical density (OD) of GAD67 bands normalized against the corresponding GAPDH in both groups of cats. The average normalized optical density of GAD67 in DR cats was significantly higher than that in the control (** p<0.01).

In order to assess if DR enhanced the GABA synthesis activities, we examined the relative abundance of the key subunit of GABA-synthesizing enzyme GAD67 in V1 from both normal control and DR cats using Western blot techniques (Fig 8). A T-test indicated that the average optical density value of GAD67 normalized against GAPDH in DR group of cats (0.594±0.048) was significantly larger than that in the control group of cats (0.393±0.052) (T-test, p<0.01).

**Fig 9:**
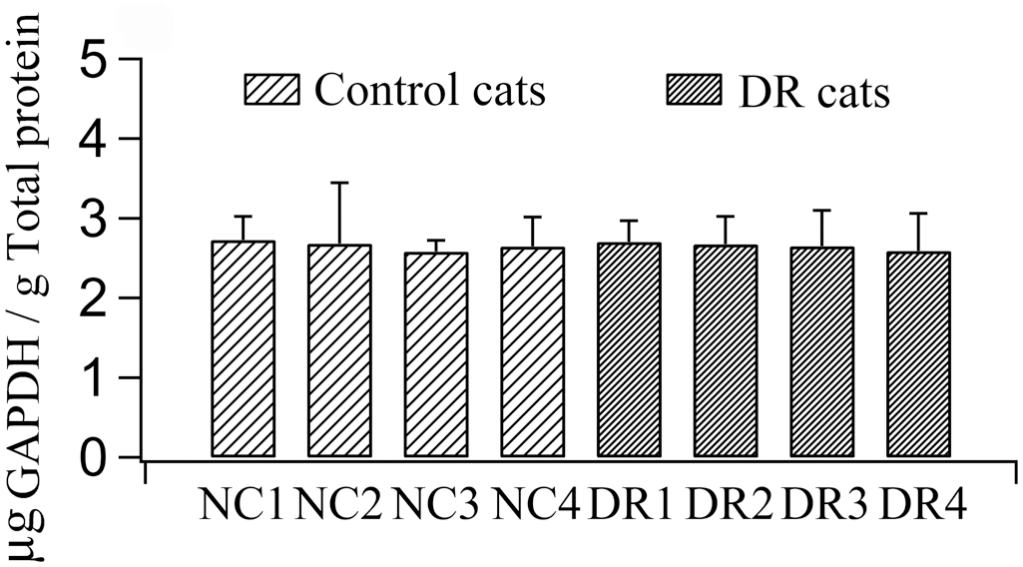
The quantity of GAPDH protein measured with enzyme linked immunosorbent assay. The quantity was expressed as a relative value of GAPDH (μg) to total proteins (g) in V1 samples of each normal control cat (NC1, NC2, NC3, NC4) and DR cat (DR1, DR2, DR3, DR4). Assays for each subject were performed in triplicate. The mean content in DR cats showed no significant difference from that in control cats (F(1,8) = 0.02, p>0.5).

An increased expression ratio of GAD67 to GAPDH could resulted from a decreased expression of GAPDH. To clarify this possibility, we quantitatively measured the content of GAPDH in the total proteins extracted from V1 tissues of each DR and normal control cat using an enzyme linked immunosorbent assay kit for rat GAPDH (S1 Text). Our results indicated that the mean proportion of GAPDH to total proteins in V1 homogenate of each control cat showed no significant difference from that of each DR cat (Group effect: F(1,24) = 0.001, p>0.5; Interaction of group and subject: F(3,24) = 0.023, p>0.5). Further, the averaged proportion of GAPDH to total proteins across all control cats exhibited no significant difference from that across all DR cats also (F(1,8) = 0.02, p>0.5) (Fig 9). Therefore, we concluded that DR resulted in an increased expression of GAD67.

## Discussion

### DR-related changes of intracotical inhibition

The present study revealed that the V1 neurons in DR cats exhibited lower spontaneous activities, lower visually-evoked responses, higher signal-to-noise ratio, and stronger orientation and motion direction selectivity than did neurons in normal control cats. These results suggest that DR reduces neuronal response amplitude but improve the function of neurons in stimulus selectivity and signal extraction from noise. These neuronal response changes were not due to a deep anesthesia level applied in DR cats because the heart rate and ECG of DR and control cats were maintained in the same normal range during electrophysiological recording, and the mean dose of anesthetic-urethane (mg/kg body weight/hr) used in DR cats (20.5±0.70) and control cats (20.8±0.59) exhibited no significant difference (p>0.1) (S3 Table). Additionally, our previous investigations demonstrated that giving as much as four times the minimum level of urethane required to anesthetize cats did not alter the degree of neuronal response selectivity for stimulus orientations and motion directions nor significantly changed the visually-driven response and spontaneous activity of V1 neurons [24, 37, 38, 53].

The response function changes of V1 neurons in DR cats could have resulted from an elevation of intracotical inhibition. Firstly, this may be due to the evidence reported in previous studies which show that raising GABAergic inhibition in the V1 through electrophoretic application of GABA and GABA_A_ receptor agonist can decrease neuronal response amplitude and increase neuronal response selectivity for stimulus orientations and motion directions, whereas lowering GABAergic inhibition by administration of GABA_A_ receptor antagonist exerts the opposite effect [23, 54–56]. Further, the changes of neuronal responsiveness observed in this study were accompanied by a significant increase of GABA immunoreaction and the expression of the key GABA-synthesizing enzyme GAD67, which were consistent with other reports that DR caused a significantly increased expression of GAD65 and GAD67 in several brain regions [31, 32] and enhanced GABA-immunoreactivity in the visual cortex [13]. These observations demonstrate that DR may enhance GABAergic inhibition and contribute to DR-related neuronal function changes. Nevertheless, we cannot exclude any contributions from other neurotransmitter systems, such as norepinephrine, which is known to affect signal-to-noise ratio of the neuronal response in different sensory modalities [57–62] although inconsistent results in this respect exist in the literature [62, 63]. Recent studies indicate that moment-to-moment fluctuations of arousal in awake mice, which is reflected by the pupil dilation that is correlated with norepinephrine effect [64], impact both the sensory evoked response and the spontaneous activity of cortical neurons [65]. In the visual cortex, a heightened arousal significantly increased the signal-to-noise ratio of visual responses and reduced noise correlations [66]. As DR in the current study caused a larger reduction in body weight (up to 21%) than previous studies, the animals could be in a state of hyper-arousal, which might cause a change of norepinephrine neurotransmitter system and thus contribute to the increased signal-to-noise ratio of V1 neurons in DR cats. Further investigations are needed to clarify this situation.

Our results differed from a previous study that reported a reduced intracortical inhibition in the visual cortex of young adult rats after a short-term (4 weeks) food restriction [17]. Reasons for this discrepancy were unclear. There are three critical differences that are worthy of attention in subsequent investigations: (1) Different species may display variations in neural plasticity. (2) Animals at different age may show different effects of DR on the inhibitory neurotransmitter systems because the excitation-inhibition balance may change with age [46, 67–69]. (3) Different types, period and level of food restriction, such as short-term and long-term, may also lead to the difference of DR effects [70]. Further studies are needed to examine all these factors.

### Mechanisms of DR-related lifespan extension

Dietary restriction, such as decreasing food intake by 20–30%, has been repeatedly reported to increase longevity and delay the onset of age-associated disease in a diverse range of species [4, 5, 9, 10, 35]. However, the mechanism that mediates the DR-related anti-aging effects remains open in debate.

Previous investigations indicate that DR-related calorie limitation is easily detected by nutrient sensors and then triggers widespread adaptive and protective response in nearly all tissues and organs [71, 72]. Several molecular pathways have been implicated in mediating the DR effects, including the adenosine monophosphate (AMP) activated protein kinase (AMPK) pathway [7, 9, 73], the target of rapamycin (TOR) pathway [7, 9, 10, 74, 75], the sirtuins pathway [7, 74, 76], CREB/Sirt1 pathway [71] and the insulin like growth factor (IGF-1)/insulin signalling pathway [7]. These pathways may interact and play important roles in mediating different aspects of the response. For example, DR-induced changes in the intracellular AMP/ATP ratio can lead to activation of AMPK, which acts to maintain cellular energy stores by switching on catabolic processes that produce ATP, while switching off anabolic processes that consume ATP. Meanwhile, the sirtuins pathway (such as Sirt1) is activated due to an increased NAD^+^/NADH levels while the TOR pathway is inhibited [71, 73], which will result in suppressed cell growth, lowered protein and lipid synthesis, reduced oxidative stress, enhanced autophagy and coordinated gene expression [71]. Although, these cooperative activities are thought to delay age-related changes and promote longevity, how they impact upon neurotransmitter systems, such as inhibitory neurotransmitter system, and retard brain aging is largely unknown.

A few of studies report that DR can stimulate the production of neurotrophic factor BDNF [11–13, 77]. In addition, activation of the AMPK pathway could mediate KA (Kainic acid)-induced BDNF expression [78], and transcription factor CREB (cAMP responsive element binding) can also trigger the expression of genes encoding BDNF and its receptor TrkB in neurons [71]. Further, there is experimental evidence showing that BDNF can facilitate establishment of GABAergic synapses [79–81], modulate GABAergic synaptic transmission [82, 83] and enhance GABA synthesis and release [84–87]. Therefore, it is likely that DR-induced enhancement of GABAergic inhibition could be mediated through the activation of nutrient-sensing pathways and/or up-regulation of neurotrophic factors, such as BDNF.

An elevation of GABAergic inhibition might contribute to lifespan extension during DR. On the one hand, DR-induced improvement of GABAergic effect can counteract functional degradation of cortical neurons caused by a compromised intracortical inhibition during aging [22–24, 27]. On the other hand, although GABAergic neurons cover only about 20% of total neurons, it is widely confirmed that GABAergic inhibition plays a critical role in shaping neuronal activities in both local neural circuits and distant brain regions, including the suppression of excitability, generation of population oscillations and regulation of precise timing of neuronal firing [88–93]. Therefore, DR-induced enhancement of GABAergic inhibition can efficiently lower the excitation and firing level of a large number of neurons during signal processing and thus may greatly reduce energy expenditure in the generation and conduction of action potentials [94, 95], which will be beneficial for maintaining energy homeostasis initiated by AMPK and other nutrient-sensing pathways during DR.

In summary, the present study demonstrated that DR resulted in a significant decrease of V1 neurons in spontaneous activity and visually-evoked response, but an evident increase in the function of visual signal detection. These neuronal response changes might be related to an enhancement of GABAergic inhibition, which would enable the brain to work efficiently at a low energy cost and thus might contribute to the maintaining of energy homeostasis during DR and the extension of lifespan.

## Supporting Information

S1 Table Weekly measure of body weight (kg) for each control cat (NC1, NC2, NC3, NC4) and DR cat (DR1, DR2, DR3, DR4) during the period of DR. 0 indicates the start time point of DR, and 1–12 represent week number of DR.
(PDF)

S2 Table Record of health index for each control cat (NC1, NC2, NC3, NC4) and DR cat (DR1, DR2, DR3, DR4) during the period of DR. BT, HR, BP and OS represent body temperature (°C), heart rate (beats/min), femoral artery contraction blood pressure (mm Hg) and blood oxygen saturation (%) respectively. 0 indicates the start time point of DR, and 1–12 represent week number of DR.
(PDF)

S3 Table The mean dose of urethane (mg/kg body weight/hr) used during electrophysiological recording. NC1, NC2, NC3 and NC4 represent normal control cats. DR1, DR2, DR3 and DR4 represent DR cats.
(PDF)

S1 Text Supplementary materials and methods. GAPDH protein content measurement with enzyme linked immunosorbent assay (ELISA).
(PDF)
