# Supplementary material for: Dietary Restriction Affects Neuronal Response Property and GABA Synthesis in the Primary Visual Cortex
Source: PLoS One. 2016 Feb 10;11(2):e0149004. doi: 10.1371/journal.pone.0149004 (PMC4749323; doi:10.1371/journal.pone.0149004)
Supplement: S1 Table — 0 indicates the start time point of DR, and 1–12 represent week number of DR. (PDF) [file pone.0149004.s001.pdf]

S1 Table

Subject	Week												
	0	1	2	3	4	5	6	7	8	9	10	11	12
NC1	3.75	3.75	3.84	3.85	3.83	3.92	3.92	3.91	3.91	3.92	3.92	3.96	3.95
NC2	3.58	3.59	3.59	3.59	3.65	3.63	3.68	3.7	3.72	3.72	3.72	3.74	3.78
NC3	3.43	3.44	3.47	3.56	3.58	3.59	3.58	3.58	3.59	3.57	3.61	3.65	3.91
NC4	3.67	3.67	3.69	3.71	3.71	3.71	3.71	3.73	3.74	3.74	3.74	3.74	3.75
DR1	3.81	3.46	3.27	3.19	3.11	3.09	3.06	3.04	3.02	3.01	3.01	3.01	3.00
DR2	3.52	3.29	3.14	3.09	3.06	3.03	3.02	3.03	3.02	3.02	3.01	3.01	3.01
DR3	3.45	3.22	3.05	2.92	2.81	2.79	2.78	2.76	2.75	2.73	2.73	2.73	2.72
DR4	3.72	3.51	3.38	3.29	3.21	3.20	3.18	3.16	3.16	3.16	3.16	3.16	3.15
