# Supplementary material for: Dietary Restriction Affects Neuronal Response Property and GABA Synthesis in the Primary Visual Cortex
Source: PLoS One. 2016 Feb 10;11(2):e0149004. doi: 10.1371/journal.pone.0149004 (PMC4749323; doi:10.1371/journal.pone.0149004)
Supplement: S2 Table — BT, HR, BP and OS represent body temperature (°C), heart rate (beats/min), femoral artery contraction blood pressure (mm Hg) and blood oxygen saturation (%) respectively. 0 indicates the start time point of DR, and 1–12 represent week number of DR. (PDF) [file pone.0149004.s002.pdf]

S2 Table

Health index	Subject	Week												
		0	1	2	3	4	5	6	7	8	9	10	11	12
BT	NC1	38.0	38.1	38.2	38.3	38.0	38.3	38.2	38.1	38	38.1	38.3	38.0	38.3
	NC2	38.1	38.1	38.4	38.5	38.1	38.1	38.3	38.2	38.0	38.3	38.1	38.4	38.0
	NC3	38.3	38.4	38.1	38.1	38.4	38.2	38.5	38.4	38.1	38.2	38.0	38.4	38.2
	NC4	38.2	38.3	38.2	38.4	38.2	38.0	38.0	38.3	38.2	38.0	38.2	38.5	38.1
	DR1	38.1	38.0	38.0	38.2	38.3	38.0	38.5	38.2	38.3	38.0	38.1	38.4	38.2
	DR2	38.4	38.1	38.2	38.5	38.0	38.1	38.2	38.3	38.5	38.0	38.4	38.1	38.0
	DR3	38.2	38.0	38.1	38.4	38.2	38.3	38.0	38.1	38.2	38.3	38.0	38.3	38.4
	DR4	38.0	37.9	38.0	38.1	38.4	38.2	38.0	38.0	38.1	38.4	38.2	38.0	38.1
HR	NC1	205	196	210	203	214	213	196	204	198	205	207	208	215
	NC2	216	213	205	208	206	190	201	221	212	203	211	213	204
	NC3	194	209	202	217	189	207	204	218	190	207	198	202	208
	NC4	208	219	207	211	206	200	205	197	216	208	204	197	211
	DR1	195	206	201	198	204	216	199	216	198	202	190	206	218
	DR2	196	203	209	205	213	210	207	200	195	203	207	218	214
	DR3	210	215	212	220	206	201	214	215	217	211	188	204	208
	DR4	203	219	207	211	214	208	209	197	208	203	212	196	201
BP	NC1	131	126	105	129	123	120	122	121	127	122	128	113	106
	NC2	126	108	104	127	128	117	114	104	109	116	123	130	117
	NC3	110	125	118	124	123	132	128	118	129	130	127	119	109
	NC4	120	119	119	122	115	109	104	105	116	121	110	112	124
	DR1	121	116	120	110	113	108	123	125	117	119	122	106	116
	DR2	126	118	115	129	130	127	132	119	122	126	130	125	117
	DR3	115	124	108	109	112	104	118	114	108	113	117	120	121
	DR4	109	117	114	102	111	124	120	115	103	103	109	112	110
OS	NC1	98.3	98.4	99.2	98.5	98.4	98.6	99.5	97.1	98.2	99.2	97.6	98.7	98.8
	NC2	99.2	97.6	98.3	99.8	98.6	97.6	97.3	96.5	97.7	98.3	96.8	99.1	99.3
	NC3	99.8	98.7	98.1	97.7	99.3	98.1	98.2	97.9	99.3	97.1	99.1	98.5	96.6
	NC4	97.6	99.1	97.4	96.5	97.9	99.2	98.1	98.4	96.9	99.5	98.2	96.7	97.5
	DR1	99.5	96.2	98.8	96.0	97.3	98.1	97.6	98.0	99.3	98.4	97.8	99.1	98.4
	DR2	98.2	97.3	96.2	97.1	96.8	97.1	98.0	99.2	98.6	97.7	98.3	97.4	98.0
	DR3	99.6	96.3	97.5	98.1	97.2	98.3	99.1	97.4	99.2	98.3	98.6	97.5	98.3
	DR4	97.8	98.1	99.4	98.2	97.5	98.2	97.3	98.7	97.4	98.4	97.8	96.8	99.2
