# Supplementary material for: Dietary Restriction Affects Neuronal Response Property and GABA Synthesis in the Primary Visual Cortex
Source: PLoS One. 2016 Feb 10;11(2):e0149004. doi: 10.1371/journal.pone.0149004 (PMC4749323; doi:10.1371/journal.pone.0149004)
Supplement: S3 Table — NC1, NC2, NC3 and NC4 represent normal control cats. DR1, DR2, DR3 and DR4 represent DR cats. (PDF) [file pone.0149004.s003.pdf]

S3 Table

Subject	Mean dose of Urethane
NC1	21.4
NC2	20.1
NC3	21.2
NC4	20.6
DR1	20.4
DR2	21.3
DR3	20.0
DR4	20.2
