# Supplementary material for: Dietary Restriction Affects Neuronal Response Property and GABA Synthesis in the Primary Visual Cortex
Source: PLoS One. 2016 Feb 10;11(2):e0149004. doi: 10.1371/journal.pone.0149004 (PMC4749323; doi:10.1371/journal.pone.0149004)
Supplement: S1 Text — GAPDH protein content measurement with enzyme linked immunosorbent assay (ELISA). (PDF) [file pone.0149004.s004.pdf]

S1 Text. GAPDH protein content measurement with enzyme linked immunosorbent assay (ELISA). We quantitatively measured the content of GAPDH in the total proteins extracted from V1 tissues of each control and DR cat using a SimpleStep ELISA kit for rat GAPDH (Suzhou Calvin Biotechnology Co. Ltd., Suzhou China). The total protein concentration in each sample was measured using Coomassie brilliant blue G-250 (Sangon Biotechnology, Shanghai, China) (See Materials and Methods 1.3.2). Assays of samples and standard GAPDH protein were simultaneously conducted according to the assay kit instructions. Briefly, 50 µl standard protein with gradient concentrations (0, 3, 6, 12, 24, 48, 96 µg/L) and 10 µl samples diluted in 40 µl dilution buffer (containing 25 mM Tris–HCl pH 7.6; 150 mM NaCl, 1% NP-40, 1% sodium deoxycholate, 0.1% SDS and protease inhibitor cocktail) were added to separate wells of a 48-well plate pre-coated with mouse anti-rat GAPDH monoclonal antibody. Then, 100 µl HRP-conjugated rabbit anti-mouse IgG (dilution 1:1,000) was added to each well, and the plate was sealed and incubated for 1 h at 37 °C. Following the incubation, all wells in the plate were thoroughly washed four times with wash buffer. Thereafter, 100 µl of TMB substrate (containing 3,3´,5,5´- tetramethylbenzidine) was added to each well, and the plate was gently mixed on a shaker and incubated in the dark for 15 min at 37°C. The chromogenic reaction was stopped by adding 50 µl of H2SO4 (2 M) to each well, and the optical density (OD) of each well was read at 450 nm with a microplate reader (Tecan, Männedorf, Switzerland). GAPDH protein concentration in each sample was calculated from the OD-concentration fitting function of standard protein, and the

GAPDH content in each sample was expressed as a proportion of GAPDH (μg) to total proteins (g). The GAPDH content in each subject was an average across assays performed in triplicate.
